# International Recommendations for the Diagnosis and Management of Patients With Adrenoleukodystrophy: A Consensus-Based Approach

**DOI:** 10.1212/WNL.0000000000201374

**Published:** 2022-11-22

**Authors:** Marc Engelen, Wouter J.C. van Ballegoij, Eric James Mallack, Keith P. Van Haren, Wolfgang Köhler, Ettore Salsano, A.S.P. van Trotsenburg, Fanny Mochel, Caroline Sevin, Molly O. Regelmann, Nicholas A. Tritos, Alyssa Halper, Robin H. Lachmann, James Davison, Gerald V. Raymond, Troy C. Lund, Paul J. Orchard, Joern-Sven Kuehl, Caroline A. Lindemans, Paul Caruso, Bela Rui Turk, Ann B. Moser, Frédéric M. Vaz, Sacha Ferdinandusse, Stephan Kemp, Ali Fatemi, Florian S. Eichler, Irene C. Huffnagel

**Affiliations:** From the Department of Pediatric Neurology/Emma Children's Hospital (M.E., W.J.C.B., I.C.H.), Amsterdam UMC, Amsterdam Leukodystrophy Center, University of Amsterdam, the Netherlands; Division of Child Neurology (E.J.M.), Department of Pediatrics, Weill Cornell Medicine/NewYork-Presbyterian Hospital, NY; Department of Neurology & Pediatrics/Lucile Packard Children's Hospital (K.P.V.H.), Stanford University School of Medicine, Palo Alto, CA 4. Department of Neurology, Leukodystrophy Clinic, University of Leipzig Medical Center, Germany; Unit of Rare Neurodegenerative and Neurometabolic Diseases (E.S.), Fondazione IRCCS Istituto Neurologico C. Besta, Milano, Italy; Department of Pediatric Endocrinology/Emma Children's Hospital (A.S.P.T.), Amsterdam UMC, University of Amsterdam, the Netherlands; AP-HP (F.M.), Department of Medical Genetics, Reference Center for Adult Neurometabolic Diseases and Leukodystrophies, and INSERM U 1127, CNRS UMR 7225, Paris Brain Institute, La Pitié-Salpêtrière University Hospital, Paris, France; Department of Pediatric Neurology/Hôpital Bicêtre Paris Sud (C.S.), France, Reference Center for Children Leukodystrophies Inserm U1127, ICM—Hôpital Pitié Salpêtrière, Paris, France; Division of Pediatric Endocrinology and Diabetes (M.O.R.), Children's Hospital at Montefiore, Albert Einstein College of Medicine of Medicine, Bronx, NY; Neuroendocrine Unit (N.A.T.), Massachusetts General Hospital, Boston, MA; Harvard Medical School (N.A.T.), Boston, MA; Division of Pediatric Endocrinology (A.H.), Department of Pediatrics, Massachusetts General Hospital, Boston, MA, and Harvard Medical School (A.H.), Boston, MA; Charles Dent Metabolic Unit (R.H.L.), National Hospital for Neurology and Neurosurgery, London, United Kingdom; Metabolic Medicine (J.D.), Great Ormond Street Hospital for Children, London United Kingdom; Department of Genetic Medicine (G.V.R.), Johns Hopkins, Baltimore, MD; Division of Pediatric Blood and Marrow Transplantation & Cellular Therapy (T.L., P.J.O.), University of Minnesota, Minneapolis; Pediatric Oncology (J.-S.K.), Hematology, Hemostaseology, University Hospital Leipzig, Germany; Pediatric Blood and Bone Marrow Transplantation (C.A.L.), Princess Maxima Center Utrecht, the Netherlands; Department of Pediatrics (C.A.L.), Wilhemina Children's Hospital, UMC Utrecht, Utrecht University, the Netherlands; Director of Pediatric Neuroimaging (P.C.), Lenox Hill Radiology and Medical Imaging Associates, New York, NY; Moser Center for Leukodystrophies (B.R.T., A.F.), Kennedy Krieger Institute, Johns Hopkins Medical Institutions, Baltimore, MD; Department of Neurogenetics (A.B.M.), Kennedy Krieger Institute, Baltimore, MD; Laboratory Genetic Metabolic Diseases (F.M.V., S.F., S.K.), Department of Clinical Chemistry and Pediatrics, Amsterdam UMC, Amsterdam Gastroenterology Endocrinology Metabolism, University of Amsterdam, the Netherlands; and Department of Neurology (F.S.E.), Massachusetts General Hospital, Boston, MA. Dr. van Ballegoij is currently at the Department of Neurology, Zaans Medisch Centrum, Zaandam.

## Abstract

Pathogenic variants in the *ABCD1* gene cause adrenoleukodystrophy (ALD), a progressive metabolic disorder characterized by 3 core clinical syndromes: a slowly progressive myeloneuropathy, a rapidly progressive inflammatory leukodystrophy (cerebral ALD), and primary adrenal insufficiency. These syndromes are not present in all individuals and are not related to genotype. Cerebral ALD and adrenal insufficiency require early detection and intervention and warrant clinical surveillance because of variable penetrance and age at onset. Newborn screening has increased the number of presymptomatic individuals under observation, but clinical surveillance protocols vary. We used a consensus-based modified Delphi approach among 28 international ALD experts to develop best-practice recommendations for diagnosis, clinical surveillance, and treatment of patients with ALD. We identified 39 discrete areas of consensus. Regular monitoring to detect the onset of adrenal failure and conversion to cerebral ALD is recommended in all male patients. Hematopoietic cell transplant (HCT) is the treatment of choice for cerebral ALD. This guideline addresses a clinical need in the ALD community worldwide as the number of overall diagnoses and presymptomatic individuals is increasing because of newborn screening and greater availability of next-generation sequencing. The poor ability to predict the disease course informs current monitoring intervals but remains subject to change as more data emerge. This knowledge gap should direct future research and illustrates once again that international collaboration among physicians, researchers, and patients is essential to improving care.

Adrenoleukodystrophy (ALD) has a variable and unpredictable clinical course.^1^ It is caused by pathogenic variants in *ABCD1* causing deficient β-oxidation of saturated very-long-chain fatty acids (VLCFAs).^2-4^ VLCFAs in plasma are a diagnostic marker.^5,6^ Patients are asymptomatic at birth but develop symptoms as the disease progresses. There are 3 core clinical syndromes: a slowly progressive myeloneuropathy (adrenomyeloneuropathy), a rapidly progressive leukodystrophy (cerebral ALD), and primary adrenal insufficiency. Women develop myeloneuropathy; men can develop all 3 syndromes.^7-9^ Currently, it is not possible to predict the individual disease course.^1^ Only supportive treatment is available for the myeloneuropathy, but early-stage cerebral ALD can be stabilized with allogeneic hematopoietic cell transplantation (HCT), and adrenal insufficiency can be treated with hormone replacement therapy. Clinical surveillance is required to monitor the emergence of treatable aspects of the disease.^1^

ALD has a birth prevalence of 1:15,000 (male patients and female patients).^10^ The number of diagnosed patients is increasing because of newborn screening and exome sequencing in clinical practice. Management of patients with ALD varies between centers because of a lack of prospective natural history studies and controlled clinical trials. Clinical practice recommendations are needed to optimize outcomes. We aimed to reach consensus among international ALD experts on best approaches to diagnosis, clinical surveillance, and treatment of patients with ALD using a modified Delphi procedure.

## Methods

Three centers of excellence for ALD (Amsterdam UMC, Massachusetts General Hospital, and Kennedy Krieger Institute) initiated this project. Based on their ALD-related expertise, 30 professionals ([pediatric] neurologists, endocrinologists, metabolic specialists, HCT experts, radiologists, and laboratory scientists) were invited. Experts were selected based on existing collaborations, recent publications, and attendance of relevant scientific meetings. ME, ICH, FSE, and AF formulated clinical questions on diagnosis, clinical surveillance, and treatment relevant to clinical care for patients with ALD. The questions were subsequently screened for omissions by all experts. Feedback from 3 patient advocacy groups (ALD Connect [USA], Alex TLC [UK], and “Volwassenen, Kinderen en Stofwisselingsziekten” [NL]) ensured that the clinical questions also addressed topics deemed relevant by patients. The advocacy groups provided comments in writing, and all suggestions on underrepresented topics were adopted.

**Figure 1:**
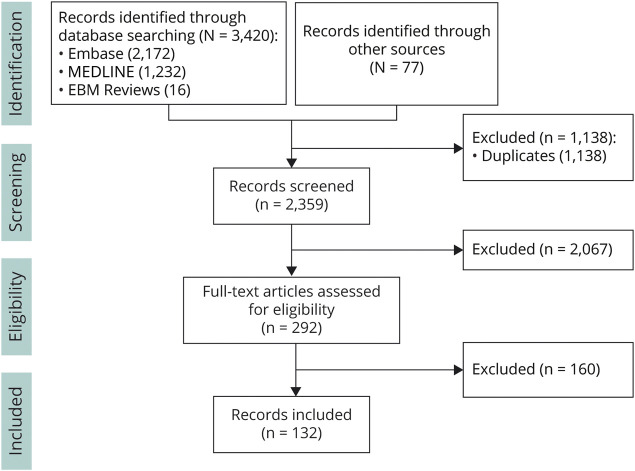
Literature Review Flowchart—Selection of Records

We used an evidence-based and consensus-based modified Delphi approach to reach consensus among the experts.^11,12^ First, a literature review (Figure 1) identified 132 relevant articles, which were mostly (small) retrospective and observational studies and thus insufficient for an isolated evidence-based approach.

**Figure 2:**
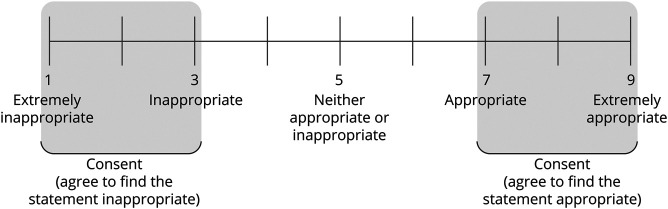
Scale Used to Define Consensus

The Delphi approach consisted of 2 questionnaire rounds and 1 consensus meeting. In the first questionnaire round, the experts rated their agreement with statements derived from the clinical questions based on a 9-point scale (1: “completely disagree” and 9: “completely agree”). Consensus was defined as follows: >80% of experts rated their “agreement” between 7 and 9 or “disagreement” between 1 and 3 (Figure 2). Statements where consensus was not reached were included in the second questionnaire round, and an anonymized overview of results derived from feedback of the first round was provided for each question alongside the experts own initial answer. Evidence tables from the literature review were provided. Experts were offered the opportunity to change their answer. The remaining statements were included in an online consensus meeting where consensus was sought by real-time discussion. The questionnaires, response numbers, and questions that did not reach consensus are listed in eAppendix 1 (links.lww.com/WNL/C362).

In this study, we summarize the recommendations that reached consensus. Twenty-eight of the 30 invited experts participated in the first questionnaire round, 26 in the second questionnaire round, and 20 in the consensus meeting. All 28 experts agreed to the final version of the recommendations. For statements concerning clinical surveillance or screening, screening was defined as clinical follow-up at predefined intervals to detect onset of symptoms or physical signs in patients who did not have any (self-reported) symptoms.

### Standard Protocol Approvals, Registrations, and Patient Consents

This study is exempt from IRB approval because conclusions were reached with available data from the literature and expert opinion (Delphi procedure). No information on individual patients is included in this article.

### Role of the Funding Source

The health consultancy agency Adelphi Values was consulted with financial support of Bluebird Bio, SwanBio Therapeutics, and Minoryx. Adelphi Values assisted the authors with the literature search and data extraction and facilitated the consensus meeting. The financial sponsors had no influence on the content of the recommendations or this article.

### Data Availability

All data on the Delphi procedure are available as supplementary data.

## Results

### Diagnosis

#### Presenting Symptoms

##### Recommendations

1. Cerebral ALD should be considered in boys and men with white matter abnormalities on brain MRI in a pattern suggestive of ALD with or without cognitive and neurologic symptoms.
2. ALD-related myelopathy should be considered in adult men and women with signs or symptoms of chronic myelopathy (gait disorder, spastic paraparesis, sphincter disturbances) with a normal MRI.
3. ALD-related adrenal insufficiency should be considered in boys and men with primary adrenal insufficiency with no detectable steroid-21-hydroxylase antibodies or other organ specific antibodies.
4. In all at-risk patients with a relative diagnosed with ALD, ALD should be considered.

Cognitive and neurologic symptoms which could indicate cerebral ALD include the new onset of attention problems, learning difficulties, onset of behavioral/mental health issues, impaired speech and vision, and progressive difficulty in walking and coordination. In boys and men with typical confluent white matter abnormalities on brain imaging—but no neurologic symptoms—cerebral ALD should still be considered as lesion development that may precede symptoms.^13^ Peripheral neuropathy is a common feature of ALD but is rarely the presenting symptom.^7,14-16^ In isolated peripheral neuropathy, other causes than ALD should be sought. Female patients with ALD remain asymptomatic in childhood and adolescence, while in adulthood, myeloneuropathy symptoms can arise.^7,14^ Cerebral ALD and primary adrenal insufficiency are extremely rare in women. Other causes of cerebral disease or adrenal insufficiency should be sought in female patients.^17^

### Diagnostic Tests

**Figure 3:**
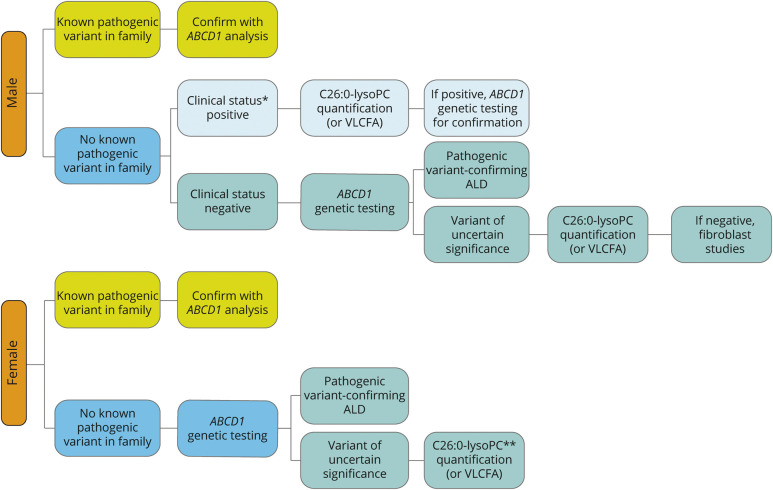
Diagnostic Algorithm Ideally, biochemical and genetic testing are combined to establish diagnosis. *Male patients are stratified by clinical status. The clinical status of ALD is positive if any symptom or sign relatable to ALD is present. This can include cerebral ALD with symptoms, cerebral ALD based on MRI abnormalities only, myeloneuropathy, or adrenal insufficiency. If no symptoms or signs relatable to ALD are present, the clinical status is negative. **Sensitivity of C26:0-lysoPC is >99%, whereas it is 85% for VLCFA. Analyze C26:0-lysoPC in case of normal plasma VLCFA. ALD = adrenoleukodystrophy; VLCFA = very-long-chain fatty acid

A diagnostic algorithm is provided in Figure 3. Genetic testing (*ABCD1* analysis) is the gold standard. For biochemical testing, plasma C26:0-lysophosphatidylcholine (C26:0-lysoPC) has superior diagnostic performance.^18^ If unavailable, fasting plasma VLCFAs (C26:0; C26:0/C22:0; C24:0/C22:0) should be obtained.^18-21^ Diagnostic algorithms are sex-specific because VLCFA may be (near) normal in women. In women, the sensitivity of VLCFAs analysis is 85%, whereas C26:0-lysoPC has a sensitivity of >99%.^18,19,22^

In symptomatic male patients with high suspicion for the diagnosis, biochemical testing should precede genetic testing. In asymptomatic male patients and all female patients, genetic testing is the first tier.

Detection of a known pathogenic *ABCD1* variant confirms the diagnosis of ALD in both men and women. De novo pathogenic variants and variants of uncertain significance (VUS) are common. It has been advocated that in patients in whom a previously unreported missense variant is identified (a VUS), confirmation of a disease-causing pathogenic variant can only be established if a clinical phenotype develops or the genetic variant is proven pathogenic in another patient displaying symptoms. That is why only in boys, men, and symptomatic women with elevated biomarkers (C26:0-lysoPC or VLCFAs), a new variant in the *ABCD1* gene is considered (likely) pathogenic.^23^

In vitro fibroblast studies are available to study the pathogenicity of a VUS in *ABCD1* and is especially recommended in asymptomatic boys and men and biomarker levels above the upper reference range of controls, but below the disease range (of the diagnostic laboratory). For women, fibroblast studies are less informative because of heterozygosity. In ambiguous cases, extended family screening may be considered although this can raise ethical questions related to testing of asymptomatic individuals. For newborn screening, positives with VUS or known benign variants in *ABCD1* and abnormal biochemistry, other peroxisomal biomarkers (i.e., plasmalogens, phytanic acid, pristanic acid, and bile acid intermediates) should be analyzed to exclude related diseases such as Zellweger spectrum disorder, *ACOX1* (or *HSD1B4*) deficiency, *CADDS*, *ACBD5* deficiency, and Aicardi-Goutières syndrome.^24^

### Treatment and Management

#### Multidisciplinary Team of Health Care Professionals

##### Recommendations

1. A central coordinator should be assigned as the case manager.
2. For male patients, a neurologist, endocrinologist, or metabolic specialist (adult or pediatric), a pediatrician, and a genetic counselor should be consulted. For female patients, this should be a neurologist, metabolic specialist, and genetic counselor.

The central coordinator may be a nurse or a (pediatric) neurologist, endocrinologist, or metabolic specialist depending on local practice and expertise. Urologists, HCT experts, rehabilitation physicians, physiotherapists, neuropsychologists, and mental health workers may be consulted. In very advanced cerebral ALD, nutritional support or speech therapy may be indicated.

**Figure 4:**
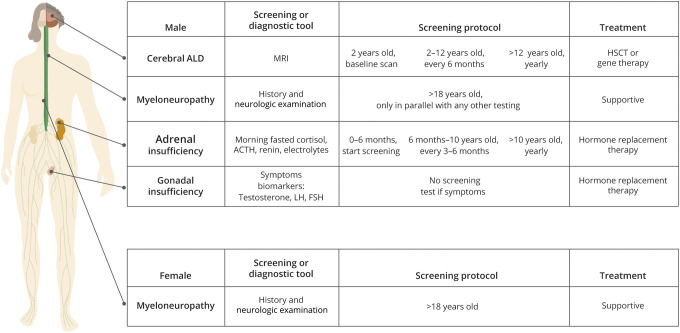
Overview of the Management of Patients With ALD ALD = adrenoleukodystrophy

An overview of the management of patients with ALD is provided in Figure 4.

### Cerebral ALD

#### Diagnosis With MRI and the Use of Gadolinium and Sedation

##### Recommendations

1. For the diagnosis of cerebral ALD, the minimum set of MR sequences is T1 (±gadolinium), T2, and FLAIR. Sedation should be used if necessary.
2. Gadolinium is indicated when a new lesion or questionable lesion is identified.
3. The interval between gadolinium administration and postcontrast T1-weighted image acquisition can influence study interpretation where shorter intervals can yield a false-negative result. Although the optimal timing is not known, based on our experience, we recommend an interval of at least 5 minutes.

Cerebral ALD causes severe disability and death.^1^ The gold standard for the diagnosis of cerebral ALD is MRI. Gadolinium enhancement just behind the leading edge of the lesion indicates active disease.^25-27^ The extent of brain involvement on MRI can be quantified with the MR severity scoring system (Loes score), ranging from 0 (no abnormalities) to 34 (severely abnormal).^28^ Recently published consensus guidelines on MRI surveillance in ALD recommend gadolinium administration for boys aged 3–12 years because the risk of developing cerebral ALD is deemed highest at this point.^29^ However, new European Medicines Agency guidelines advocate restricted gadolinium use.^30^ Therefore, our panel recommends to restrict gadolinium use to characterize newly identified or questionable lesions with 2 exceptions: (1) if sedation is required, to prevent the need for additional anesthesia exposure associated with a repeat scan, and (2) if real-time review of MR imaging is unavailable and the logistics of an additional hospital visit for gadolinium administration and repeat imaging are challenging.

### Screening for Cerebral ALD

#### Recommendations

1. All boys and men with ALD should be screened for cerebral ALD, including in the absence of neurologic or cognitive symptoms.
2. A baseline MRI scan should be obtained at age 2 years. Between 2 and 12 years, male patients should be screened every 6 months. From age 12 years, screening should be yearly.
3. We do not recommend routine screening for cerebral ALD in girls and women.

The screening protocol is presented in Figure 4. We do not recommend routine screening for cerebral disease in female patients.^17,31,32^ Irrespective of screening strategies, an MRI should be performed as soon as possible in all patients who develop potential signs or symptoms of cerebral ALD during follow-up.

The risk of developing cerebral disease is highest in childhood but remains present throughout life.^1,29,33-39^ The age for the baseline MRI was set at 2 years because cerebral ALD before the age of 3 years is rare, and MRI scans obtained before the age of 2 years are difficult to interpret because of incomplete myelination. Clinicians may choose to obtain an MRI at earlier time points based on clinical presentation and experience.^10^

For adult men, MRI surveillance should continue as long as HCT is a therapeutic option (maximum allowable age for HCT varies and was not subject to consensus here), although extended surveillance may be requested by individual patients for prognostic purposes. In patients with presumed arrested (nonprogressive and nonenhancing) MRI abnormalities, imaging should initially be repeated after 3 months to confirm that lesions are nonenhancing and stable. If stable, the frequency can be expanded to 6 months and thereafter adhering to abovementioned age-specific screening recommendations.^13,25,39,40, e1^ Scans may be repeated more frequently in individual ambiguous cases.

### Alternative Methods for Screening and Diagnosis of Cerebral Disease

#### Recommendations

1. Nonimaging biomarkers for the diagnosis of cerebral ALD are not indicated outside of a research setting.
2. No consensus was reached on the implementation of neuropsychological testing as a screening tool for cerebral ALD.

Differentiating active from arrested white matter lesions can be challenging.^13,25,39,40,e1^ Several biomarkers (i.e., plasma neurofilament light, chitotriosidase and cytokine levels) have been studied for this purpose but require additional validation.^e2-e5^ Neuropsychological changes may precede MRI changes,^e6,e7^ and therefore, neuropsychological screening could be a useful tool to screen for cerebral ALD in parallel to MRI.

### Cerebral ALD Treatment

#### Recommendations

1. Transplantation eligibility should be determined by an ALD transplantation expert.
2. Eligibility criteria are not exclusive. In general, boys are considered eligible for transplantation when they have demyelination with gadolinium enhancement (MR severity score [Loes score] ≤ 9) and a neurologic function score of 0 or 1 and adult men when they have demyelinating lesions with gadolinium enhancement and no or few neurocognitive impairment.
3. Genetically transduced autologous stem cell transplantation (gene therapy) should be considered (if available) in boys if allogeneic donor options are poor.

Allogeneic hematopoietic cell transplantation (HCT) is the standard treatment for cerebral ALD and can halt progression.^34-36,38,e8-e13^ Outcome is poor in advanced disease (Loes score >9 and/or neurologic function score >1).^e14^ In men, severe spinal cord disease (Expanded Disability Status Scale score >6) and bilateral internal capsule involvement are associated with poor survival.^e15^

In boys, autologous hematopoietic stem cell transplantation after ex vivo lentiviral gene therapy has been studied as a safer alternative. Long-term safety data are not yet available. ^e15-e17^ Currently, this therapy is not available for routine care. Treatment for boys or men with advanced disease or progressive lesions without gadolinium enhancement should only be considered after careful evaluation in experienced centers.

### Non–Cerebral-Related Treatment Effects of Transplantation on Myeloneuropathy and Adrenal Insufficiency

HCT is unlikely to affect myeloneuropathy or adrenal insufficiency, but data are limited.^e18,e19^ For the very new ex vivo gene-corrected autologous transplantation approach, there are no data.

#### Lifestyle Management

##### Recommendation

1. Male patients should be counseled on the possible association between head injury and onset of cerebral disease so that they can make an informed lifestyle choice.

Severe head trauma has been reported as possible trigger for cerebral ALD.^e20-e22^ Definitive proof on causality is not available.

#### Myeloneuropathy

Screening for and follow-up of myeloneuropathy:

##### Recommendations

1. History and neurologic examination should be used to diagnose myeloneuropathy.
2. Asymptomatic men and women should only be screened for symptoms or physical signs of myeloneuropathy in parallel with other testing.
3. For men and women with myeloneuropathy, we recommend yearly follow-up. For men, coordinating annual visit with annual brain MRI may improve convenience and compliance.

Virtually all men and most women with ALD eventually develop symptoms and signs of myeloneuropathy.^8,33,e23-e25^ Extensive counseling on specific symptoms should be considered at the time of diagnosis of ALD. This helps prevent the incorrect attribution of unrelated symptoms to the ALD diagnosis and promotes early identification of ALD-related symptoms. Follow-up can also be used to update patients on developments in the field. Men and women presenting with new symptoms should be evaluated for myeloneuropathy. The use of standardized clinimetric tests and electrophysiologic testing remains restricted to research settings.^7,14,16,17,31,e24,e26,e27^

### Treatment of Myeloneuropathy

#### Recommendations

1. Treatment is supportive and should be aimed at reducing pain (with pharmaceuticals such as pregabalin or gabapentin) and spasticity (with spasmolytics like baclofen) and maintaining functional ability and quality of life.
2. In addition to routine neurologic care, referral to a rehabilitation specialist, continence care specialist, or pain management specialist/team may be considered.

No consensus was reached on recommendations for invasive spasticity treatments (i.e., intrathecal baclofen therapy, selective dorsal rhizotomy) or urinary incontinence.

### Adrenal Insufficiency

#### Screening for Adrenal Insufficiency

##### Recommendations

1. All boys and men, but not girls and women, should be routinely screened for adrenal insufficiency with early morning cortisol and adrenocorticotropin hormone (ACTH) measurements.
2. Screening for adrenal insufficiency should be initiated in the first 6 months of life. Then, patients should be screened every 3–6 months before the age of 10 years and yearly thereafter. Screening should be performed parallel to MRI where possible.
3. All patients in whom symptoms suggestive of adrenal insufficiency manifest should undergo prompt evaluation for adrenal insufficiency to identify and prevent an adrenal crisis. If the patient is in crisis, a random cortisol and ACTH level measurement is sufficient (provided that serum specimens are drawn before glucocorticoid administration); if mildly symptomatic, early morning fasted cortisol and ACTH measurement is preferred.
4. All patients who are screened for adrenal insufficiency, or after diagnosis of adrenal insufficiency, should also be screened for mineralocorticoid deficiency with plasma renin and serum electrolytes.
5. All patients in whom symptoms suggestive of mineralocorticoid deficiency manifest should undergo prompt evaluation with plasma renin and serum electrolytes.

Primary adrenal insufficiency is common in male patients with ALD, but rare in women.^33,e28,e29^ Screening in adult patients should continue irrespective of age because it is inexpensive, and early diagnosis offers a large potential health benefit.

Adrenal insufficiency is characterized by low or normal early morning cortisol levels with high levels of ACTH.^1,33^ For diagnosis, random cortisol and ACTH can be used when early morning measurement is not an option; however, when the results are ambiguous, patients should be retested with early morning fasted cortisol and ACTH. Synthetic ACTH stimulation testing can be restricted to ambiguous cases or when early morning fasted cortisol and ACTH are difficult to obtain. Besides glucocorticoid deficiency, some patients also develop mineralocorticoid deficiency (hypoaldosteronism).^33,e30,e31^

### Treatment of Adrenal Insufficiency

#### Recommendations

1. If adrenal insufficiency is present, we recommend glucocorticoid replacement therapy by an endocrinologist.
2. Mineralocorticoid replacement therapy should not be initiated based on symptoms alone but should also take into account plasma renin and serum electrolyte abnormalities.
3. Routine evaluation of bone health with dual energy x-ray absorptiometry measurements in boys with adrenal insufficiency and glucocorticoid replacement therapy is not recommended.
4. No consensus was reached on the evaluation of bone health in men.

Patients with ambiguous results such as abnormal ACTH with normal or borderline cortisol values on stimulation testing should be managed per existing guidelines.^e30,e31^ Long-term glucocorticoid replacement therapy has been associated with impaired bone health.^e32^

### Gonadal Insufficiency

#### Screening for Gonadal Insufficiency

##### Recommendations

1. Boys and men should not be screened for gonadal insufficiency.
2. If symptoms manifest, gonadal insufficiency should be evaluated with biochemical testing (early morning testosterone, LH, FSH). In boys, delayed progression to puberty could indicate gonadal insufficiency (testicular volume <4 mL and/or no signs of puberty by the age of 14 years).

Abnormal hormone levels indicating gonadal insufficiency were described in boys and men with ALD.^e33-e36^ The clinical relevance of these findings remains unclear. For instance, erectile dysfunction may reflect primary gonadal insufficiency or can be caused by spinal cord disease.^1^ Testing (gonadotrophins and testosterone measured in the early morning) is only recommended if there are symptoms.^e34,e37^

#### Treatment of Gonadal Insufficiency

##### Recommendation

1. Treatment of gonadal insufficiency is restricted to endocrinologists.

Transdermal testosterone/long-acting testosterone ester injections can be used to treat gonadal insufficiency if levels are below normal and symptoms are present.

### Dietary Therapy

#### Recommendation

1. Data to support the efficacy of Lorenzo's oil as a disease-modifying treatment in patients with ALD is insufficient.

Lorenzo's oil (oleic acid [C18:1] and erucic acid [C22:1]) in combination with a low-fat diet reduces plasma C26:0 levels to (near) normal values in most of the patients, but controlled clinical trials showing improved outcome are lacking.^16,31,e38-e41^

### Additional Management Recommendations

1. All patients who are diagnosed with ALD should be informed about the option of family screening.
2. Patients who wish to conceive must be informed regarding the potential benefits of preimplantation (preimplantation genetic diagnosis; oocyte donation) and prenatal (noninvasive fetal sex determination in maternal plasma; invasive prenatal testing) diagnostic options.
3. We recommend classifying female patients with an *ABCD1* pathogenic variant as “asymptomatic/presymptomatic” or “symptomatic women with ALD” and to refrain from using terms as heterozygotes or carriers.

## Discussion

We provide recommendations on diagnosis, clinical surveillance, and treatment of ALD. Our consensus-based approach among ALD experts across the world allowed us to formulate recommendations despite limited scientific evidence. Still, on some topics, consensus could not be reached (for instance, the exact timing of adrenal testing in the first year of life). Furthermore, this approach is at risk for bias because of panelist selection and repeat review. We aimed to include all relevant specialists with experience in the field of ALD, but this group is small, and the clinical experience of panelists varied from smaller regional to larger international cohorts with more than 100 patients. Experts were instructed to withhold from voting on recommendations outside their field of expertise, but no additional correction was applied for differences in clinical experience. Moreover, consensus was defined as >80% agreement. Therefore, some panelists did not agree with specific recommendations although consensus was reached. The screening protocol for cerebral ALD and lifestyle management are examples of topics that remain subject of discussion. Nonetheless, this guideline addresses a need in the ALD community because of the worldwide increase in diagnoses and the number of presymptomatic individuals because of newborn screening and improved diagnostic procedures. Moreover, we highlight knowledge gaps that can direct future research and illustrates that international collaboration among physicians, researchers, and patients is essential to improve care.

### Panel 1: Search Strategy and Selection Criteria

We searched Embase, MEDLINE, and Evidence-Based Medicine Reviews for relevant publications on diagnosis, clinical surveillance, and treatment of patients with ALD. No medical librarian was involved. The search was limited to full-text availability in English and a publication date between 1990 to the year 2019. The search terms used were “adrenoleukodystrophy” or “x-linked adrenoleukodystrophy” or “X-ALD.” For the syntax of the search, refer to page 9 of the literature review report provided as supplementary data (eMethods, links.lww.com/WNL/C364). In addition, we conducted a gray literature search of internet-based sources and extended the search with snowballing. The gray literature search included GeneReviews, UpToDate, ClinicalKey, The International Leukodystrophy Association (or Global Leukodystrophy Initiative), ALD.info, and the Society for the Study of Inborn Errors of Metabolism. To provide up to date published data that were not captured in full-text publications, we also included recent conference proceedings and summits, that is, the ALD Connect annual meeting, the European Pediatric Neurology Society meeting, the American Society of Gene and Cell Therapy annual meeting, the United Leukodystrophy Foundation conference, and the American Academy of Neurology annual meeting. All articles on ALD involving human participants were included, and nonhuman (animal) studies were excluded. Detailed inclusion and exclusion criteria can be found in the literature review report (eMethods, links.lww.com/WNL/C364). Two reviewers of Adelphi Values (A.V.) independently screened all abstracts and where applicable concomitant full text for their eligibility. After completion, the results were reviewed and compared by a third senior reviewer (A.V.), to resolve discrepancies and reach consensus on inclusion in the literature review. The results of this consensus process and the results of the data extraction are summarized in the data extraction form that is available as eAppendix 2 (links.lww.com/WNL/C363). The study quality was assessed using the Newcastle-Ottawa Scale for Assessing the Quality of Nonrandomized Studies in Meta-analysis, and the results are available as eTable 1 (links.lww.com/WNL/C366).

### Panel 2: Summary of Recommendations

#### Presenting Symptoms—When to Consider ALD

1. In boys and men with confluent white matter abnormalities on brain MRI in a pattern suggestive of ALD with or without cognitive and neurologic symptoms;
2. In adult men and women with symptoms and signs of chronic myelopathy with a normal MRI;
3. In boys and men with primary adrenal insufficiency with no detectable steroid-21-hydroxylase antibodies or other organ specific antibodies;
4. In all at-risk patients with a relative diagnosed with ALD.

### Diagnostic Tests

A diagnostic algorithm is provided in Figure 3.

### Screening, Diagnosis, and Treatment of Cerebral ALD

7-12. Screen all boys and men for cerebral ALD with MRI, including in the absence of neurologic or cognitive symptoms. Screening frequency is discussed in Figure 4. Gadolinium is indicated when a new lesion or questionable lesion is identified, or sedation is used.

15-17. To treat cerebral ALD, consult an ALD transplantation expert who can determine allogeneic or genetically transduced autologous stem cell transplantation eligibility.

#### Screening, Diagnosis, and Treatment of Myeloneuropathy

19. Use history and neurologic examination to diagnose myeloneuropathy.
20. Solely screen apparently asymptomatic men for symptoms or physical signs of myeloneuropathy in parallel to any other testing.
21. Schedule yearly follow-up for men and women with myeloneuropathy
22. Treatment is supportive. Aim treatment at reducing pain and spasticity and maintaining functional ability and quality of life.

### Screening, Diagnosis, and Treatment of Adrenal Insufficiency

24-28. Screen all boys and men for adrenal insufficiency with early morning cortisol, ACTH, plasma renin, and serum electrolytes. If symptoms suggestive of adrenal insufficiency manifest, evaluate adrenal insufficiency promptly to prevent an adrenal crisis.

29-30. Consult a (pediatric) endocrinologist for glucocorticoid replacement therapy when adrenal insufficiency is present. Do not initiate mineralocorticoid replacement therapy based on symptoms alone but also take into account plasma renin and serum electrolyte abnormalities.

### Dietary Therapy

36. Data to support the efficacy of Lorenzo's oil as a disease-modifying treatment in patients with ALD are insufficient.

## Glossary

ACTH: adrenocorticotropin hormone
ALD: adrenoleukodystrophy
VUS: variants of uncertain significance
